# Fear of Falling among Older Patients Admitted to Hospital after Falls in Vietnam: Prevalence, Associated Factors and Correlation with Impaired Health-Related Quality of Life

**DOI:** 10.3390/ijerph17072493

**Published:** 2020-04-06

**Authors:** Long Hoang Nguyen, Giang Thu Vu, Giang Hai Ha, Cuong Tat Nguyen, Hai Minh Vu, Tien Quoc Nguyen, Tung Hoang Tran, Kiet Tuan Huy Pham, Carl A. Latkin, Bach Xuan Tran, Roger C.M. Ho, Cyrus S.H. Ho

**Affiliations:** 1 Center of Excellence in Behavioral Medicine, Nguyen Tat Thanh University, Ho Chi Minh City 700000, Vietnam; long.coentt@gmail.com (L.H.N.);; 2 Center of Excellence in Evidence-based Medicine, Nguyen Tat Thanh University, Ho Chi Minh City 700000, Vietnam; giang.coentt@gmail.com; 3 Institute for Global Health Innovations, Duy Tan University, Da Nang 550000, VietNam; nguyentatcuong@duytan.edu.vn; 4 Faculty of Pharmacy, Duy Tan University, Danang 550000, Vietnam; 5 Faculty of Medicine, Duy Tan University, Danang 550000, Vietnam; 6 Department of Trauma and Orthopaedic, Thai Binh Medical University Hospital, Thai Binh 410000, Vietnam; vuminhhai777@gmail.com; 7 Department of Environment Health-Faculty of Public Health, Thai Binh University of Medicine and Pharmacy, Thai Binh 410000, Vietnam; ngqtienytb@gmail.com; 8 Institute of Orthopaedic and Trauma Surgery, Vietnam—Germany Hospital, Hanoi 100000, Vietnam; tranhoangtung.vd@gmail.com; 9 Institute for Preventive Medicine and Public Health, Hanoi Medical University, Hanoi 10000, Vietnam; phamhuytuankiet_vkt@fpt.vn (K.T.H.P.); bach.ipmph@gmail.com (B.X.T.); 10 Bloomberg School of Public Health, Johns Hopkins University, Baltimore, MD 21205, USA; carl.latkin@jhu.edu; 11 Department of Psychological Medicine, Yong Loo Lin School of Medicine, National University of Singapore, Singapore 119228, Singapore; 12 Institute for Health Innovation and Technology (iHealthtech), National University of Singapore, Singapore 119077, Singapore; 13 Department of Psychological Medicine, National University Hospital, Singapore 119074, Singapore; cyrushosh@gmail.com

**Keywords:** fear of falling, health-related quality of life, EQ-5D-5L, older people, Vietnam

## Abstract

Fear of falling (FOF) diminishes older people’s independence in daily activities, as well as causes serious health and economic consequences. This study examined the prevalence of FOF in older patients hospitalized due to fall-injuries, its effect on health-related quality of life (HRQOL), and its associated factors. We conducted a cross-sectional study in seven hospitals in Thai Binh, Vietnam. FOF was assessed using a single close-ended question. HRQOL was evaluated by the EQ-5D-5L instrument. Multilevel logistic regression and Tobit regression models were utilized. The prevalence of FOF in 405 older patients admitted to hospitals after fall injuries was 88.2%, with a mean EQ-5D index and EQ-VAS of 0.34 (SD = 0.38) and 61.6 (SD = 15.2), respectively. Factors associated with FOF included living alone (OR = 0.13, 95%CI = 0.04; 0.50.,), history of eye diseases (OR = 4.12; 95%CI = 1.91; 8.89), and experiencing psychological distress (OR= 3.56, 95% CI = 1.05; 12.00). After adjusting for confounders, the EQ-5D index in the FOF group reduced by 0.15 points (Coef. = −0.15; 95%CI= −0.24; −0.05) compared to that of non-FOF group. Our study shows that FOF had an independent negative relationship with HRQOL of patients. Improving knowledge about fall prevention in patients and caregivers could reduce the burden of falls in older people.

## 1. Introduction

Fear of falling (FOF) is a pervasive psychological problem in elder people, and is both a major cause and consequence of falls [1,2]. FOF is referred to as “a lasting concern about falling that can lead to an individual avoiding activities that he/she remains capable of performing” [3]. Falls and fall-related injuries, particularly recurrent falls, increase the vulnerability of older adults to FOF [4,5,6,7]. Moreover, FOF may reduce mobility and balance capacities, which could result in falls or recurrent falls [8,9]. In addition, FOF lessens the confidence of the elderly in performing daily activities, limits their physical functionalities, and increases their social dependence, medication use, and healthcare cost [4,10,11,12]. The prevalence of FOF in the elderly varies across settings from 12% to 65% in community samples, 28–95% among older people who ever experienced falls in their lifetime [6,7,10,13,14], and 30.0–62.8% in older populations admitted to the hospital [15,16,17]. Thus, thoroughly understanding the circumstance of FOF in specific settings as well as its associated factors are critically important to develop fall prevention strategies in the elderly population.

Along with the need for epidemiological evidence, investigating how FOF influences health outcomes such as health-related quality of life (HRQOL) is also essential, as it enables one to evaluate the performance of fall interventions in different health aspects [18]. Indeed, HRQOL has been advocated to become an integral indicator for interventions aiming to diminish the burden of falls and their consequences [19,20]. Previous studies indicated that FOF was associated with poorer HRQOL regarding physical, psychological and social perspectives [11,14,21,22]; and the decrease of FOF was related to the enhancement of HRQOL [23]. Short-form 12 (SF-12) and EuroQol-5 Dimensions (EQ-5D) are two recommended HRQOL instruments, especially the latter one [18,24,25,26]. The EQ-5D tool is a short, simple instrument, free of charge, allowing the computation of a health utility index that can be used in further health economic evaluations [27].

Vietnam has been undergoing rapid growth of their aging population compared to other countries [28]. The percentage of older residents aged 60 and over will reach 25% of the general population by 2049 [28]. This trend will cause a great burden to the healthcare system unless appropriate strategies to prevent falls in older people are taken. To date, only one study was performed in Vietnamese community-dwelling older adults, which found that 64% of the sample had a high level of FOF [29]. However, the study was not conducted among older patients hospitalized due to falls, as well as the effect of FOF on patients’ HRQOL. Although several previous studies in the world examined the FOF rates in hospitalized older individuals, they did not investigate factors associated with FOF in this population [15,16,17]. Therefore, this study aims to measure the prevalence of FOF in older patients admitted to the hospitals due to fall-injuries, determine associated factors with FOF, and examine the influence of FOF on HRQOL.

## 2. Materials and Methods

### 2.1. Study Design and Sample

This was a secondary analysis using data from a multi-site cross-sectional study, which was performed in seven hospitals of Thai Binh province, Thai Binh, Vietnam from August 2018 to February 2019. Methods for this study are described elsewhere [30,31]. In short, 405 patients were conveniently recruited from Thai Binh Provincial General Hospital, Kien Xuong, Quynh Phu, Tien Hai, Thai Thuy, Dong Hung, and Hung Ha District General Hospitals. They met the eligibility criteria including: (1) being aged 60 years or over; (2) using ambulatory or inpatient services due to fall injuries during the study period; (3) agreeing to participate in the study and giving their written or verbal informed consents, and (4) not having any cognitive impairment that could affect their ability to undertake face-to-face interviews within 15 min. The last criterion was confirmed by the physicians who directly treated the patients. A total of 430 patients were invited to participate, and 405 patients accepted to be study participants (response rate 94.2%). The reasons for refusal included being tired, not wanting to be disturbed, and refusal from caregivers.

### 2.2. Measurement

Data collection was carried out by using a structured questionnaire via face-to-face interviews. Data collectors consisted of undergraduate medical students studying in the Thai Binh University of Medicine and Pharmacy. They were intensively trained in a two-day workshop session about the purpose of this study, the structure of the questionnaire, and the interview communication skills. The interviewers were also involved in a pilot study by interviewing ten patients to understand the interview procedures and to assure high quality data collection. The questionnaire was piloted and revised based on patients’ and experts’ feedback before being approved by the research team and hospitals’ leaderboard. Physicians and nurses in the hospitals helped data collection team to approach eligible patients. Patients were introduced the study in brief as well as the benefits and drawbacks of participation. After agreeing to be enrolled in the study, they signed the written informed consent or verbal informed consent was obtained if they did not want to disclose their personal information. Variables of interest are described below.

#### 2.2.1. Fear of Falling

In this study, we used a single close-ended question, “Are you afraid of falling?” with a “Yes/No” answer. This approach has been used widely in the past [9,32,33]. In fact, we considered using other scales such as “Survey of Activities and Fear of Falling in the Elderly” (SAFE) or “Fall Efficacy Scale-International” (FES-I). However, our sample was older adults hospitalized due to falls who were more likely to experience severe pain or major psychological problems; hence, using such long and complicated instruments was not feasible. Moreover, our preferred approach is simple, straightforward, and easy to acquire responses from participants [34].

#### 2.2.2. Health-Related Quality of Life

Information about HRQOL of older patients was gathered by employing the EuroQol-5 Dimensions-5 Levels (EQ-5D-5L). This is a generic preference-based instrument that evaluates HRQOL in five dimensions comprising mobility, self-care, usual activity, pain/discomfort, and anxiety/depression. Patients can answer each dimension by selecting one of five response options from “no problem” to “extreme problem” [35]. There are 3125 health states as a result of the combinations of responses. Each health state has a corresponding health utility index, the so-called EQ-5D index, which is derived from a Vietnamese cross-walk value set [36]. Patients selecting the option “no problem” was classified into the “No problem” group; otherwise, they were classified into the “Having problem” group. This tool has been widely used in Vietnam [37,38,39,40,41,42]. Moreover, we utilized the EQ-Visual Analogue Scale (EQ-VAS) to measure current self-rated health, with a score ranging from 0 “The worse health state that you can imagine” to 100 “The best health state that you can imagine”.

#### 2.2.3. Other Measurement

Along with FOF and HRQOL, patients were asked to report their sociodemographic characteristics (age, gender, education level, marital status, living location, living arrangements, and caregivers), and clinical characteristics (type of patients, current diagnosed morbidities, current medication use, mobility status, loss of sensation in hand/foot, history of eye diseases). The Kessler- Psychological Distress Scale 6 items (K6) was used to evaluate the psychological distress. This instrument has 6 items with a range of scores from 0 to 24, with higher scores reflecting a higher level of psychological distress. Patients having a score of 6 points or more were categorized as “Having psychological distress” [43].

### 2.3. Statistical Analysis

We performed data analysis by using Stata software version 15.0. Descriptive statistics were conducted with Chi-squared, Fisher’s exact and Mann–Whitney tests to evaluate the differences in the prevalence of FOF according to different groups. We also examined the differences in HRQOL dimensions, EQ-5D index and EQ-VAS between the FOF group and non-FOF group. Multilevel Logistic Regression, with patient level and hospital level, was conducted to identify the factors related to FOF. Meanwhile, as the EQ-5D index and EQ-VAS are censored data, multiple Tobit regression (or Censored regression) was used to explore the effect of FOF on the EQ-5D index and EQ-VAS. A *p*-value of less than 0.05 was considered statistically significant.

### 2.4. Ethical Approval

The Institutional Review Board of Thai Binh University of Medicine and Pharmacy reviewed and approved the study protocol (Code: 7641/HDDD).

## 3. Results

Sociodemographic characteristics of older patients have been presented elsewhere [30,31]. Overall, the prevalence of FOF in older patients hospitalized after fall injuries was 88.2%. Table 1 depicts the prevalence of FOF according to sociodemographic characteristics. The rate of FOF was significantly higher in patients who were female (91.4%), living with children (95.1%), and had children as a caregiver (94.9%) (*p* < 0.05) compared to other groups.

Table 2 shows that the prevalence of FOF was remarkably lower in patients who were physically active (85.3%), without loss of sensation (84.0%), history of eye diseases (80.1%) or psychological distress (85.3%), as well as those that received a fall prevention guideline previously (83.2%), compared to other groups (*p* < 0.05).

In unadjusted models (Table 3), living with children, having a history of eyes diseases and experiencing psychological distress were associated with higher risk of FOF. Meanwhile, living alone was negatively associated with FOF. However, in multivariate models, after adjustments, only having a history of eye disease (OR = 4.12; 95% CI = 1.91; 8.89), or experiencing psychological distress (OR = 3.56, 95% CI = 1.05; 12.00), increased the odds of reporting FOF in older patients hospitalized due to fall injuries, while living alone (OR = 0.13, 95% CI = 0.04; 0.50) decreased the odds of reporting FOF.

Distributions of EQ-5D dimensions as well as EQ-5D index and EQ-VAS are shown in Table 4. The mean EQ-5D index and EQ-VAS in the FOF group were 0.34 (SD = 0.38) and 61.6 (SD = 15.2), respectively, which were significantly lower than the non-FOF groups (EQ-5D index = 0.56 (SD = 0.22), and EQ-VAS = 66.9 (SD = 13.8)) (*p* < 0.05). Regarding dimensions, the rates of patients having problems in self-care and anxiety/depression in the FOF group were significantly higher than the non-FOF group (*p* < 0.05).

In the unadjusted regression model, the results show a 0.22 point decrement in the EQ-5D index (Coef. = −0.22; 95%CI = −0.33; −0.11) and a 5.23 point decrement in the EQ-VAS (Coef. = −5.23; 95%CI = −0.77; −0.69) in the FOF group compared to the non-FOF group. However, after adjusting for other covariates (Model 2-6), the significant decrement was only found in the EQ-5D index, with a decrease of 0.15 points (Coef. = −0.15; 95%CI = −0.24; −0.05) in comparison with the EQ-5D index of the non-FOF group. (Table 5)

## 4. Discussion

Our study contributes to the current literature on the pervasiveness of FOF as well as the influence of FOF on HRQOL in older people. The findings of this study indicated a substantial prevalence of FOF among older patients admitted to the hospital after falls. Moreover, this study shows a great effect of FOF on the decrement of the EQ-5D index, implying the urgent need for interventions to prevent FOF in older people.

The rate of FOF in our sample was remarkable with 88.2% reporting FOF. The possible explanation is that all of our sample was patients who were using inpatient or outpatient services during the data collection period. This result is comparable to other studies performed in older people experiencing falls previously, such as in Japan (92%) [44] and Brazil (80.8% in those having ≥3 falls) [45], but higher than hospitalized older populations in Iran (36.4%) [17], Germany (62.8%) [16] and Australia (30%) [15]. A prior systematic review estimated that the prevalence of FOF in general older adults (including both older people with and without fall experience) was from 20.8% to 85% [1]; hence, it is understandable that our rate was significantly high. Moreover, all patients in our study were hospitalized due to fall injuries, which was one of main risk factors for FOF [6,7,10,13,14,17]. Whereas, the studies in Iran, Germany and Australia recruited patients with different health conditions [15,16,17]. In addition, in this study, we used a single item with dichotomous choices (Yes/No) to measure the FOF. This approach is simple, clear and direct and supposed to be sensitive in indicating the change overtime [46]. However, it might overestimate the actual FOF rate because it could not evaluate the fear degree in specific conditions, which is a major drawback when comparing to other instruments such as the Modified Survey of Activities and Fear of Falling Scale, Falls Efficacy Scales, or Fear of Falling Questionnaire [13,47,48]. Nonetheless, these tools might not be appropriate to use in our setting given that our sample was hospitalized after fall injuries. They were more likely to experience severe pain or major psychological problems; hence, using such long and complicated instruments was not feasible. Huang et al. compared four approaches to measure FOF and concluded that a single item should be used when having only a short time for interview, as well as avoiding missing data as much as possible [16].

In the descriptive analysis, we found that living with children or having children as a caregiver and moving with aids were associated with FOF. These relations were in line with previous studies in the community, which indicated that the prevalence of FOF increased among females, and people with a higher degree of morbidity, and poorer physical health [29,32,45]. Women were more likely to be concerned with their health condition than men, particularly older women [49]. Indeed, women are vulnerable to several musculoskeletal diseases such as osteoporosis or bone fracture [50], which increases their susceptibility to falling as well as worsens the fall’s consequences [29]. Therefore, they were more likely to express FOF. Meanwhile, in Vietnam, men are treated as “strong” persons that do not show fear easily. In other words, they are afraid of being stigmatized if they express their fear, leading to underreporting FOF in men [29,51]. Additionally, living alone decreased the risk of FOF. These phenomena can be explained by the fact that in Vietnamese culture, if older people suffer from injuries or are hospitalized due to falls, they may be unintentionally discouraged from social independence by their spouses or child caregivers, given that it possibly increases the risk of falls. This may result in diminishing older people’s self-efficacy for fall prevention, as well as rising FOF [51]. After adjusting for other covariates, this association was maintained, suggesting that further interventions for the group vulnerable to FOF should be given attention. 

Our finding was in line with previous studies that older patients with psychological problems had more likelihood of reporting FOF [29,32,34,45]. Furthermore, people with a history of eye disease were also more likely to suffer from FOF. Indeed, both conditions may diminish people’s confidence in daily activities as well as their self-efficacy, which are important predictors of FOF [29,52,53]. The study found the marginal association that patients who did not remember whether they received fall prevention guidelines before were more likely to experience FOF. In the literature, acquiring sufficient knowledge and perception about fall prevention are critical factors to protect older people from falls [54,55]. Thus, providing counseling and guideline about fall prevention to older patients might be necessary to help them avoid FOF.

In this study, after adjusting for potential confounders, the influence of FOF on the decrement of HRQOL was significant, which aligned with previous studies [22,27]. Specifically, the FOF group scored 0.15 points lower in the EQ-5D index than the non-FOF group. This result is even substantial when comparing to other diseases in Vietnamese older people [31,37]. The difference in EQ-5D index between non-FOF and FOF might be attributable to the difference in the prevalence of having problems in self-care and anxiety/depression. These findings were consistent with previous studies as well as the results discussed above as FOF was closely correlated with psychological problems as well as low self-efficacy, less autonomy, and social dependence [4,10,11,12]. Otherwise, our results show non-significant differences between non-FOF and FOF groups regarding the rate of having problems in mobility, usual activity or pain/discomfort. This might be due to the fact that our sample was older people admitted to the hospital due to fall injuries, and these problems were the consequences of fall injuries, particularly pain/discomfort. In addition, the sample size of the non-FOF group was relatively small, which might hinder the statistical power to identify the significant difference between non-FOF and FOF groups.

Meanwhile, regarding EQ-VAS, in the univariate model, we found that fearful patients reported a significantly lower score than those without FOF, which was congruent with other studies [56]. However, after adjustment, no association was found between EQ-VAS and FOF. This phenomenon could be because EQ-VAS might only be used to reflect the perception of patients about their health rather than their actual health conditions [57], which was affected by other factors such as age and sex. A prior study indicated that, even among disease-free people, females or people with higher age tended to report lower EQ-VAS scores compared to males and younger ones, respectively [58]. Thus, since FOF does not directly affect the physical health of patients, the effect of FOF on their psychological health might not be sufficiently large to reveal a significant association. Meanwhile, the EQ-5D index was calculated via responses of five dimensions, which might reflect the actual HRQOL, especially among those with specific diseases or illnesses [37,57].

Some methodological issues should be noted in this study. First, several variables regarding physical functioning such as the strength and balance test, muscle test, or walking speed test were not collected, which might be important predictors for FOF. Furthermore, other variables such as severity of injuries or typology of fall were not collected. Other studies should be conducted to examine the associations between these variables and FOF. Second, we did not employ any validated scales to measure FOF. Further studies should fill this knowledge gap. Third, we employed a cross-sectional design, which does not allow us to conclude the causal associations between FOF and related factors, as well as FOF and the decrement of HRQOL. Moreover, information such as history of falls or eye diseases was self-reported data, which possibly resulted in recall bias. Finally, generalizability of our results might not be obtained due to the nature of the convenience sampling method and the limited sample size and study settings.

## 5. Conclusions

Our study shows a substantial prevalence of FOF in older patients hospitalized after falls in Vietnam. Moreover, FOF had an independent negative relationship with the HRQOL of patients. Education programs to improve knowledge about fall prevention in both patients and caregivers should be beneficial for reducing the burden of falls in older people.

## Figures and Tables

**Table 1:** Prevalence of fear of falling according to sociodemographic characteristics of respondents.

Characteristics	*n*	%	Prevalence of FOF (%)	*p*-Value
**Total**	405	100.0	88.2	
**Age groups**				
60–69	196	48.4	84.2	0.06
70–79	118	29.1	91.5	
≥80	91	22.5	92.3	
**Gender**				
Male	162	40.0	83.3	0.01
Female	243	60.0	91.4	
**Education**				
<High school	349	86.2	88.5	0.54
≥High school	56	13.8	85.7	
**Marital status**				
Single	131	32.3	88.6	0.86
Having spouse/partner	274	67.7	88.0	
**Living location**				
Urban	32	7.9	90.6	0.65
Rural	373	92.1	87.9	
**Living arrangements**				
Spouse	235	58.0	86.8	<0.01
Alone	17	4.2	64.7	
Children	122	30.1	95.1	
Others	31	7.7	83.9	
**Caregiver**				
Spouse	212	52.4	85.4	<0.01
Children	158	39.0	94.9	
Other	35	8.6	74.3	

**Table 2:** Prevalence of FOF according to clinical characteristics.

Characteristics	*n*	%	Prevalence of FOF (%)	*p*-Value
**Number of morbidities**				
0	99	24.4	86.9	0.09
1	160	39.5	90.6	
2	102	25.2	82.4	
≥3	44	10.9	95.5	
**Mobility status**				
Move independently	293	72.4	85.3	0.02
Move with aids	39	9.6	97.4	
Move with assisted devices	73	18.0	94.5	
**Loss of sensation in hand/foot**				
No	238	58.8	84.0	<0.01
Yes	167	41.2	94.0	
**History of eye diseases**				
No	186	45.9	80.1	<0.01
Yes	219	54.1	95.0	
**Receiving fall prevention guideline**				
Yes	143	35.3	83.2	0.04
No	191	47.2	89.5	
Not remember	71	17.5	94.4	
**Psychological distress**				
No	299	73.8	85.3	<0.01
Yes	106	26.2	96.2	
**History of falls in the last 12 months**				
1 fall	241	59.5	86.3	0.26
2 falls	68	16.8	88.2	
≥3 falls	96	23.7	92.7	
**Type of patient**				
Inpatient	151	37.3	90.7	0.22
Outpatient	254	62.7	86.6	

**Table 3:** Associated factors with FOF.

Characteristics	Unadjusted Model		Adjusted Model	
	OR	95% CI	OR	95% CI
Living arrangements				
Spouse	ref		ref	
Alone	0.33 *	0.11; 0.99	0.13 *	0.04; 0.50
Children	2.56 *	1.02; 6.44	1.78	0.67; 4.73
Others	1.05	0.35; 3.09	0.64	0.20; 2.01
Number of morbidities				
0	ref		ref	
1	2.24	0.94; 5.29	1.80	0.73; 4.46
2	1.29	0.52; 3.19	0.69	0.25; 1.84
≥3	3.87	0.81; 18.53	2.32	0.44; 12.3
History of eye diseases				
No	ref		ref	
Yes	4.64 *	2.20; 9.78	4.12 *	1.91; 8.89
Psychological distress				
No	ref		ref	
Yes	3.36 *	1.14; 9.89	3.56 *	1.05; 12.00
Receiving fall prevention guideline				
Yes	ref		ref	
No	1.08	0.52; 2.27	1.38	0.63; 3.02
Not remember	2.56	0.82; 8.05	3.31	0.99; 11.10

* *p* < 0.05.

**Table 4:** Percentage of patients having problems in each domain of EQ-5D-5L according to FOF status.

Characteristics	Non-FOF (*n* = 48)		FOF (*n* = 357)		Total		*p*-Value
	*n*	%	*n*	%	*n*	%	
Having problems in mobility	39	81.3	306	85.7	345	85.2	0.41
Having problems in self-care	41	85.4	341	95.5	382	94.3	<0.01
Having problems in usual activity	43	89.6	338	94.7	381	94.1	0.16
Having problems in pain/discomfort	48	100.0	354	99.2	402	99.3	0.52
Having problems in anxiety/depression	40	83.3	340	95.2	380	93.8	<0.01
	**Mean**	**SD**	**Mean**	**SD**	**Mean**	**SD**	
EQ-5D-5L index	0.56	0.22	0.34	0.38	0.37	0.37	<0.01
EQ-VAS	66.9	13.8	61.6	15.2	62.3	15.1	0.03

**Table 5:** Tobit regression models to identify the association between FOF status and EQ-5D index and EQ-VAS.

Model	EQ-5D Index			EQ-VAS		
	Coef.	95% CI		Coef.	95% CI	
Model 1: Unadjusted (Univariate regression model)	−0.22 *	−0.33	−0.11	−5.23 *	−0.77	−0.69
Model 2: Model 1 + age + sex	−0.18 *	−0.29	−0.07	−3.73	−8.22	0.75
Model 3: Model 2 + education + marital status + living location	−0.18 *	−0.29	−0.07	−3.80	−9.26	0.65
Model 4: Model 3 + living arrangement	−0.18 *	−0.29	−0.07	−3.96	−9.42	0.50
Model 5: Model 4 + mobility status + number of morbidities + psychological distress	−0.17 *	−0.28	−0.07	−4.06	−8.49	0.38
Model 6: Model 5 + history of falls + type of patient	−0.15 *	−0.24	−0.05	−3.31	−7.43	0.81

* *p* < 0.05.
